# Exploring new benefits of vitamin A: alleviating hypoxia-induced mitochondrial stress and mitophagy in the gills of adult grass carp (*Ctenopharyngodon idellus*)

**DOI:** 10.1186/s40104-025-01309-3

**Published:** 2025-12-16

**Authors:** Hua Cheng, Lin Feng, Pei Wu, Yang Liu, Yaobin Ma, Hongmei Ren, Xiaowan Jin, Xiaoqiu Zhou, Weidan Jiang

**Affiliations:** 1 Animal Nutrition Institute, Sichuan Agricultural University, Chengdu, 611130 China; 2 Fish Nutrition and Safety Production University Key Laboratory of Sichuan Province, Sichuan Agricultural University, Chengdu, 611130 China; 3 Key Laboratory of Animal Disease-Resistance Nutrition, Ministry of Education, Ministry of Agriculture and Rural Affairs, Key Laboratory of Sichuan Province, Chengdu, 611130 China

**Keywords:** Gills, Grass carp (*Ctenopharyngodon idellus*), Hypoxic stress, Mitochondrial stress, Mitophagy, Vitamin A

## Abstract

**Background:**

Hypoxia is a pervasive challenge in aquaculture that poses a significant threat to aquatic organisms. Since fish cannot synthesize vitamin A endogenously, it must be supplied through diet, and it plays a vital role in supporting fish stress resistance. This study aimed to investigate the protective effects of VA on the gills of adult grass carp (*Ctenopharyngodon idella*) against hypoxia and to elucidate the underlying mechanisms.

**Methods:**

Six experimental diets with graded VA levels (375, 862, 1,614, 2,099, 2,786, and 3,118 IU/kg) were fed to grass carp (initial weight: 726 ± 1.2 g) for 60 d. After the trial, 24 fish per treatment were selected, divided equally into normoxic and hypoxic groups, fasted for 24 h, and then subjected to a 96-h acute hypoxic challenge.

**Results:**

The results demonstrated that VA supplementation mitigated hypoxia-induced damage in gill tissue, as evidenced by histological examination. Furthermore, VA alleviated oxidative stress, as indicated by reduced levels of lactate (LD), lactate dehydrogenase (LDH), reactive oxygen species (ROS), protein carbonyl (PC), and malondialdehyde (MDA). Further investigations indicated that VA alleviated mitochondrial stress, potentially through suppressing the canonical UPR^mt^ axis while activating both the UPR^mt^ sirtuin axis and the UPR^IMS^/Erα axis. VA also modulated mitochondrial mass via multiple mechanisms, including the promotion of mitochondrial biogenesis, maintenance of dynamics by stimulating fusion and reducing fission, and inhibition of mitophagy. The suppression of mitophagy likely involved downregulating both the Pink1/Parkin-dependent pathway and the Hif1a-Bnip3 pathway. Taken together, these adaptations suggested an essential role for VA in preserving mitochondrial homeostasis. Based on the quadratic regression analysis of ROS and MDA levels from the hypoxic group, the estimated VA requirements for adult grass carp were 2,013 and 2,056 IU/kg diet, respectively.

**Conclusions:**

In summary, this study provided the first evidence that VA conferred protective effects against hypoxia-induced gill damage in grass carp.

**Graphical Abstract:**

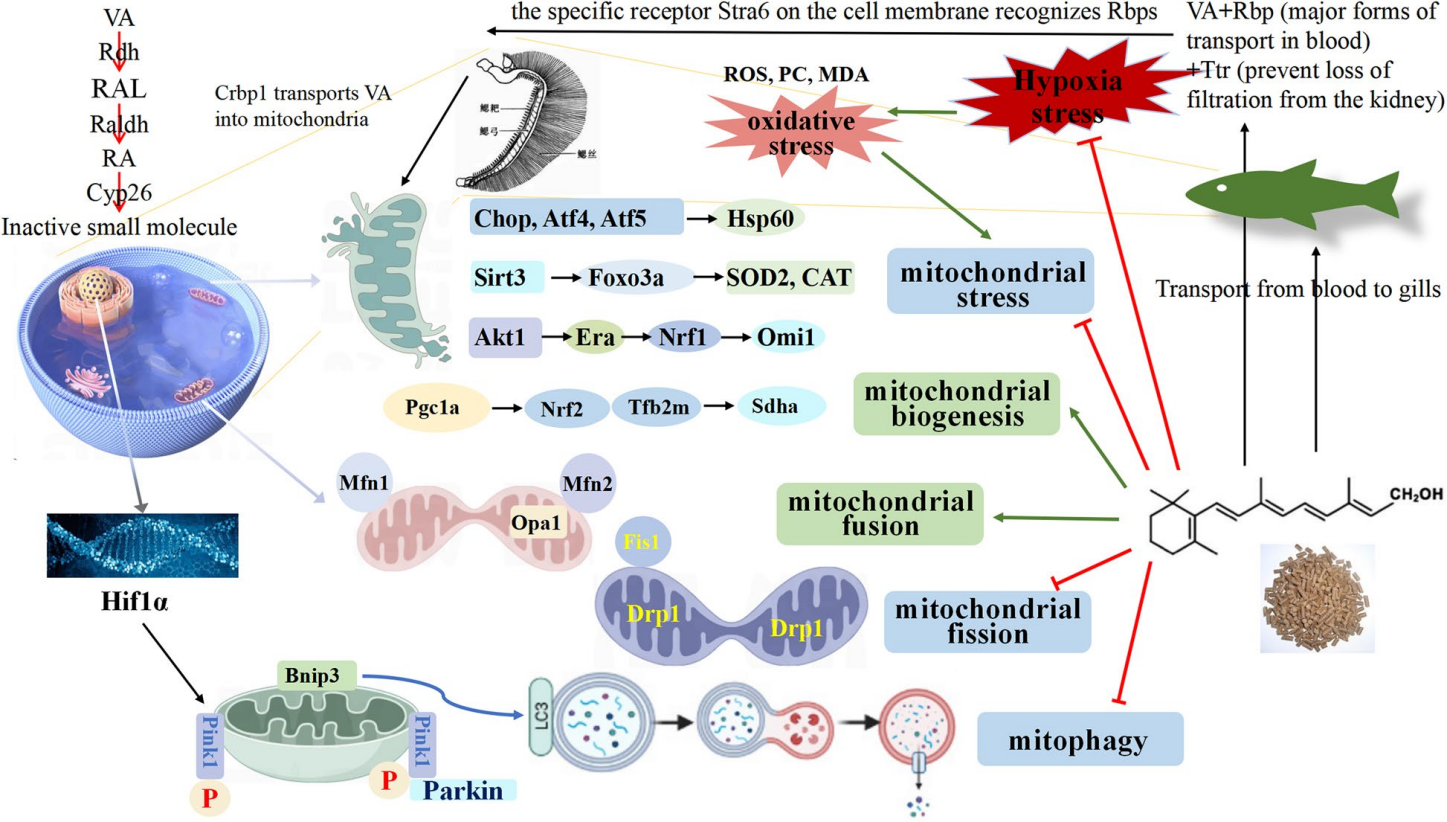

**Supplementary Information:**

The online version contains supplementary material available at 10.1186/s40104-025-01309-3.

## Introduction

Intensive aquaculture practices and global warming have contributed to an increased frequency of hypoxic events in aquatic farming systems [1]. Hypoxia adversely affects aquatic organisms by impairing locomotor capacity, retarding growth, and, under severe conditions, causing mass mortality [2]. The structural and functional integrity of gills is critical for the adaptation of fish to hypoxic conditions [3]. Previous study has demonstrated that hypoxic stress induced gill tissue damage and disrupted mitochondrial dynamics in grass carp (*Ctenopharyngodon idella*) [4]. Vitamins are commonly supplemented in aquaculture diets to mitigate such stressors. Vitamin A, the first identified fat-soluble vitamin, plays essential roles in maintaining vision, cellular differentiation, antioxidant defense, immune enhancement, and growth promotion [5]. Earlier research from our laboratory has shown that VA improved growth performance and gill structural integrity in grass carp [6, 7]. Other study has shown that VA alleviated oxidative stress caused by dietary oxidized lipids in Siberian sturgeons (*Acipenser baeri*) [8]. In addition, retinoic acid, a metabolite of VA, has been shown to attenuate hypoxic-ischemic brain injury in rats by suppressing apoptosis [9], and combined supplementation with vitamins A and C reduced renal cell damage following hypoxia-reperfusion [10]. Nevertheless, the potential role of VA in enhancing hypoxia resistance in fish remains underexplored.

Mitochondria are highly sensitive to a diverse range of environmental stressors. Mitochondrial stress refers to the cellular response to stimuli that impair mitochondrial function, including oxidative stress and mitochondrial DNA damage [11]. Different axes of the mitochondrial unfolded protein response (UPR^mt^) are activated in response to mitochondrial protein misfolding: the canonical UPR^mt^ pathway alters the expression levels of C/EBP homologous protein (Chop) and activating transcription factor 4 (Atf4); the UPR^mt^ sirtuin axis upregulates manganese superoxide dismutase (SOD2) and catalase (CAT) to enhance antioxidant defenses; and the UPR^IMS^/Erα axis induces nuclear respiratory factor 1 (Nrf1) [12]. However, the role of VA in modulating mitochondrial stress in animals remains unexplored. Previous research has shown that VA reduces interleukin-1β (IL-1β) levels in the intestinal tract of grass carp [7]. Interestingly, IL-1β has been found to upregulate Atf4 protein expression in human placental choriocarcinoma cells [13]. Additionally, VA supplementation enhanced the activity of SOD2 and CAT in grass carp gills [6]. Collectively, these findings suggest a potential involvement of VA in mitochondrial stress pathways, though its precise role and mechanisms under hypoxic conditions warrant further investigation.

Mitochondrial stress is thought to trigger reprogramming of mitochondrial biogenesis and metabolism [14]. Mitochondrial quality control (MQC) involves processes such as mitochondrial biogenesis, fission, fusion, and mitophagy [15]. A key regulator of mitochondrial biogenesis is the proliferator-activated receptor gamma coactivator 1α (Pgc1α)/Nrf1 [16]. Although the effect of VA on Pgc1α/Nrf1 in fish has not been investigated, VA has been shown to upregulate Pgc1α protein levels in sheep muscle [17].

Mitochondrial homeostasis is regulated by fusion and fission. Fusion involves mitochondrial fusion proteins (Mfn1 and Mfn2), and optic atrophy protein 1 (Opa1), while fission depends on dynamin-related protein 1 (Drp1) and receptors such as mitochondrial fission protein 1 (Fis1) [18]. Nevertheless, studies on the effects of VA on mitochondrial fusion and fission in fish remain limited. Previous research has shown that VA upregulates NF-E2-related factor 2 (*nrf2*) in the gills of grass carp [6], and *nrf2* activation leads to Drp1 degradation and increased Mfn2 expression [19]. These findings suggest a potential role for VA in modulating mitochondrial dynamics. However, further investigation is needed to clarify its exact function and underlying mechanisms.

Mitophagy is closely linked to mitochondrial fusion and fission. Through the segregation of damaged mitochondrial regions, fission facilitates the engulfment of impaired organelles by autophagosomes, thereby contributing to mitochondrial quality control [20]. The degradation of these components is primarily mediated by mitophagy via the PTEN-induced putative kinase 1 (Pink1)/Parkin-dependent and Bcl-2 adenovirus E1 B 19 kDa interacting protein 3 (Bnip3)/Nix pathways [21]. However, the effects of VA on mitophagy in fish have not been directly studied. Evidence from human epithelial cells indicates that VA can reduce mitophagy [22]. Furthermore, VA has been shown to increase arginine content in the muscle of grass carp [23]. Notably, arginine suppressed the elevated protein levels of hypoxia-inducible factor 1α (Hif1α) induced by cerebral ischemia/reperfusion in rats [24], and *hif1α* is known to upregulate the expression of the *bnip3* [25]. Collectively, these findings suggest that VA may attenuate mitophagy under hypoxic stress. Nevertheless, the exact mechanisms involved remain unclear and warrant further investigation.

Grass carp is the most productive freshwater fish species in China [26]. However, these fish are susceptible to illness and mortality under hypoxic conditions [27], a risk that may be mitigated by VA supplementation. Thus, the present study aims to investigate whether dietary VA can alleviate mitochondrial stress in the gills of grass carp exposed to hypoxic conditions. To our knowledge, this is the first study to explore this specific aspect in fish. The results are expected to provide a theoretical basis for developing vitamin-based nutritional strategies to enhance stress resistance in farmed fish.

## Materials and methods

### Ethics statement

All experimental procedures were approved by the Animal Ethics Committee of Sichuan Agricultural University (Protocol No. CH-2022214054, China) and were conducted in accordance with its relevant guidelines and regulations.

### Design of hypoxia stress experiment and sample collection

The formula of this study is listed in Table S1. Retinyl acetate (500,000 IU/g; form of VA addition) was purchased from Livestock Science Company in Dayi County, Chengdu City, Sichuan Province, China. After a one-month acclimation period, 600 grass carp (sourced from Zhong jiang, China) with an average initial weight of 726 ± 1.2 g were selected and randomly distributed into 24 circular tanks (1 m diameter × 1.25 m height) each stocked with 25 fish. The growth trial followed a completely randomized design with six dietary treatments (375, 862, 1,614, 2,099, 2,786, and 3,118 IU/kg VA), each replicated four times. The fish were fed to apparent satiation four times daily (07:00, 11:00, 15:00, and 19:00 h). A collection tray (50 cm in diameter) was placed at the bottom of each tank to recover uneaten feed. The rearing water was replaced once daily. Throughout the experimental period, water quality was maintained as follows: temperature 29 ± 2 °C, pH 7.3–7.8, and dissolved oxygen (DO) at 6.0 mg/L. The feeding trial lasted for 60 d. Following a 24-h fasting period at the conclusion of the growth trial, a hypoxia stress test was conducted. A total of 12 experimental groups were established: 6 hypoxic groups and 6 corresponding normoxic control groups, each containing 12 fish. Throughout the 96 h stress period, DO was maintained at 6 mg/L in the normoxic groups and at a constant level of 1 mg/L in the hypoxic groups. No feeding occurred during the stress test.

After the hypoxic stress experiment, grass carp were anesthetized using ethyl 3-aminobenzoate methanesulfonate (50 mg/L). Blood samples were collected from the caudal vein, and serum was separated by centrifugation at 1,610 × *g* for 15 min at 4 °C, then stored at −20 °C. Subsequently, the fish were euthanized by a sharp blow to the head. Gill tissues were sampled from eight randomly selected fish per treatment group. A portion of the gills was snap-frozen in liquid nitrogen and stored at −80 °C; the remaining samples were fixed in either 4% paraformaldehyde or 2.5% glutaraldehyde for further analysis.

### Biochemical analysis

Gill tissue samples were weighed and homogenized in ice-cold saline. The homogenate was centrifuged at 1,610 × *g* for 10 min at 4 °C, and the supernatant was collected and kept on ice for subsequent analysis. The following parameters were measured according to the manufacturer’s instructions (Nanjing Jiancheng Bioengineering Institute, China): activities of SOD2, CAT, and succinate dehydrogenase (SDH); levels of reactive oxygen species (ROS), protein carbonyl (PC), malondialdehyde (MDA), and ATP in gill tissue; as well as serum lactate content and lactate dehydrogenase (LDH) activity. VA concentration in gill tissue was determined using a commercial ELISA kit (Dumabio, China). All procedures were performed on ice. Details of the assay kits are provided in Table S2.

### Hematoxylin-eosin (H&E) staining

Gill tissues previously fixed in 4% paraformaldehyde were processed using standard histological methods. Briefly, samples were dehydrated through a graded series of ethanol, embedded in paraffin, and sectioned at a thickness of 5 μm. The sections were then stained with hematoxylin and eosin and examined under a Nikon light microscope at 200× magnification.

### Transmission electron microscope (TEM)

Gill samples were post-fixed in 1% osmium tetroxide after initial fixation in 2.5% glutaraldehyde. They were then dehydrated through a graded acetone series and embedded in Epon812 resin. Semi-thin sections were stained with methylene blue for preliminary examination. Ultrathin sections were cut using a diamond knife, stained with uranyl acetate and lead citrate, and examined under a JEM-1400-FLASH transmission electron microscope (JEOL, Japan).

### Immunofluorescence staining

Paraffin-embedded sections were prepared from gill tissue previously fixed in 4% paraformaldehyde. The sections were deparaffinized, rehydrated, treated with 3% H₂O₂ to inactivate endogenous peroxidases, and subjected to antigen retrieval. Non-specific binding was blocked using 5% goat serum in BSA. Sections were then incubated overnight at 4 °C with the following primary antibodies: Estrogen receptor α (Era) (1:1,000, Huabio, China), Yin-Yang 1 (Yy1) (1:200, Huabio, China), Translocase of outer mitochondrial membrane 20 (Tomm20) (1:500, Immunoway, USA), and Chop (1:200, ABclonal, China). After washing, a fluorescent secondary antibody (1:1,000) was applied and incubated in the dark. Nuclei were counterstained with DAPI, autofluorescence was quenched, and sections were mounted. Imaging was performed using an inverted fluorescence microscope (Leica DMI4000 B, Germany), and fluorescence intensity was quantified with ImageJ 1.53 k software. Detailed antibody information is provided in Table S3.

### Real-time quantitative PCR analysis

The gill RNA was extracted with RNAiso Plus. The quantity and quality of RNA were evaluated by 1% agarose gel electrophoresis and 260:280 nm spectrophotometry. The TaKaRa kit reverse-transcribed the RNA into cDNA. For quantitative analysis of the fluorescence, the SYBR^®^ Prime Script™ RT-PCR Kit II was used. The qRT-PCR was detected with a quantitative thermal cycler QuantStudio™ 5 (Thermo Fisher Scientific, USA). The β-actin gene was used as an internal control, and relative gene expression levels were calculated using the 2^−ΔΔCT^ method [28]. All primer sequences used in this study are listed in Table S4.

### Western blot experiment

Western blot analysis was performed according to established laboratory protocols [29]. Total protein was extracted from gill tissue using RIPA lysis buffer supplemented with PMSF (Beyotime, China). Protein concentration was determined using a BCA assay kit (Vazyme, China). The target proteins were separated with SDS-PAGE and transferred onto PVDF membranes. The membranes were blocked with BSA, washed with TBST, and incubated with primary antibodies at 4 °C overnight (≥ 17 h). Subsequently, membranes were incubated with a horseradish peroxidase (HRP)-conjugated goat anti-rabbit secondary antibody (1:70,000; Santa Cruz, China) for 2 h at room temperature. Protein bands were visualized using an ECL kit (Affinity, China) and quantified with ImageJ software. Detailed antibody information is provided in Table S3.

### Statistical analysis

All data were analyzed using SPSS statistical software and are presented as mean ± standard deviation (SD). The effects of VA were assessed by one-way ANOVA followed by Duncan’s multiple range test. Differences between hypoxic and normoxic conditions at the same VA level were compared using independent samples *t*-tests. A two-way ANOVA was employed to evaluate the interaction between VA and DO levels. Differences were considered statistically significant at *P* < 0.05.

## Results

### Blood biochemical indices

**Fig. 1:**
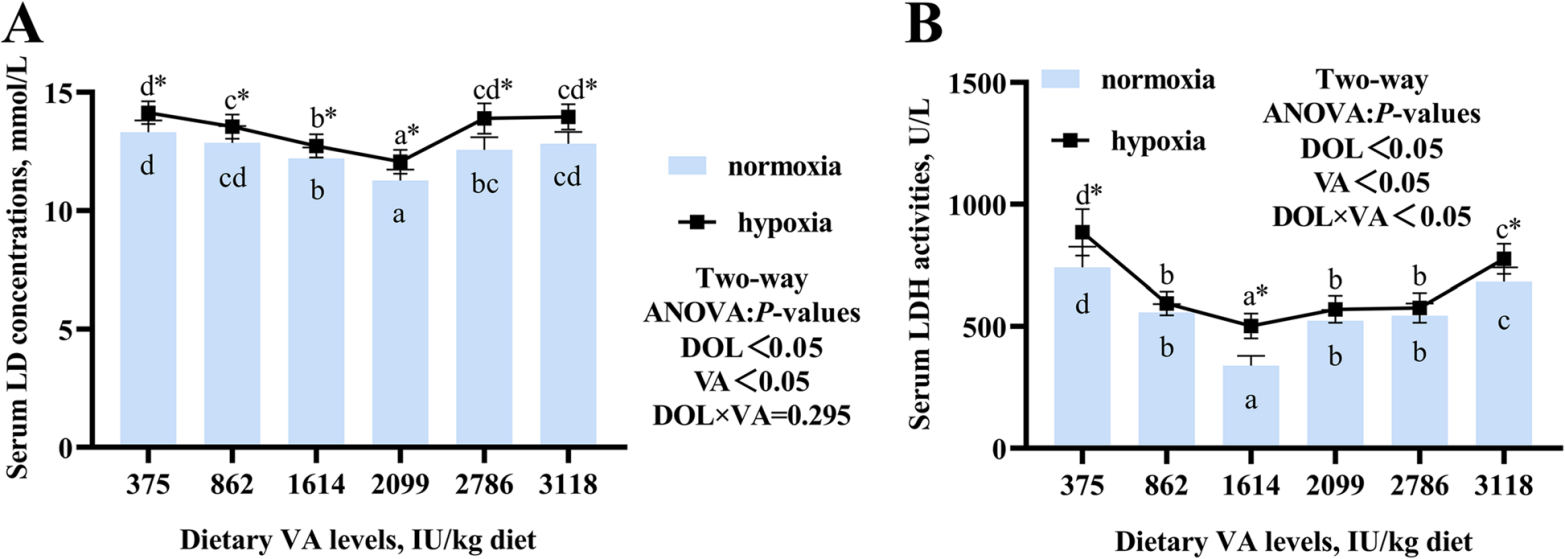
Effects of vitamin A on serum biochemical parameters in grass carp (*Ctenopharyngodon idellus*) following hypoxic stress. **A** Serum lactic acid (LD) concentration (*n* = 8). **B** Serum lactate dehydrogenase (LDH) activity (*n* = 8). Values are means ± standard deviation. Different letters indicate significant differences between VA treatments at the same dissolved oxygen level (*P* < 0.05). “*” indicates significant differences between normoxic and hypoxic groups at the same VA level (*P* < 0.05)

Serum LD and LDH in the normoxic and hypoxic groups reached lowest values at dietary VA levels of 2,099 and 1,614 IU/kg, respectively (Fig. 1). Hypoxia significantly increased both serum LD and LDH compared to normoxic conditions at the same VA levels (*P* < 0.05). Two-way ANOVA revealed that serum LDH was significantly influenced by VA, DO, and their interaction (*P* < 0.05). In contrast, the interaction between VA and DO did not have a significant effect on LD levels (*P* > 0.05).

### VA transport in gills

**Fig. 2:**
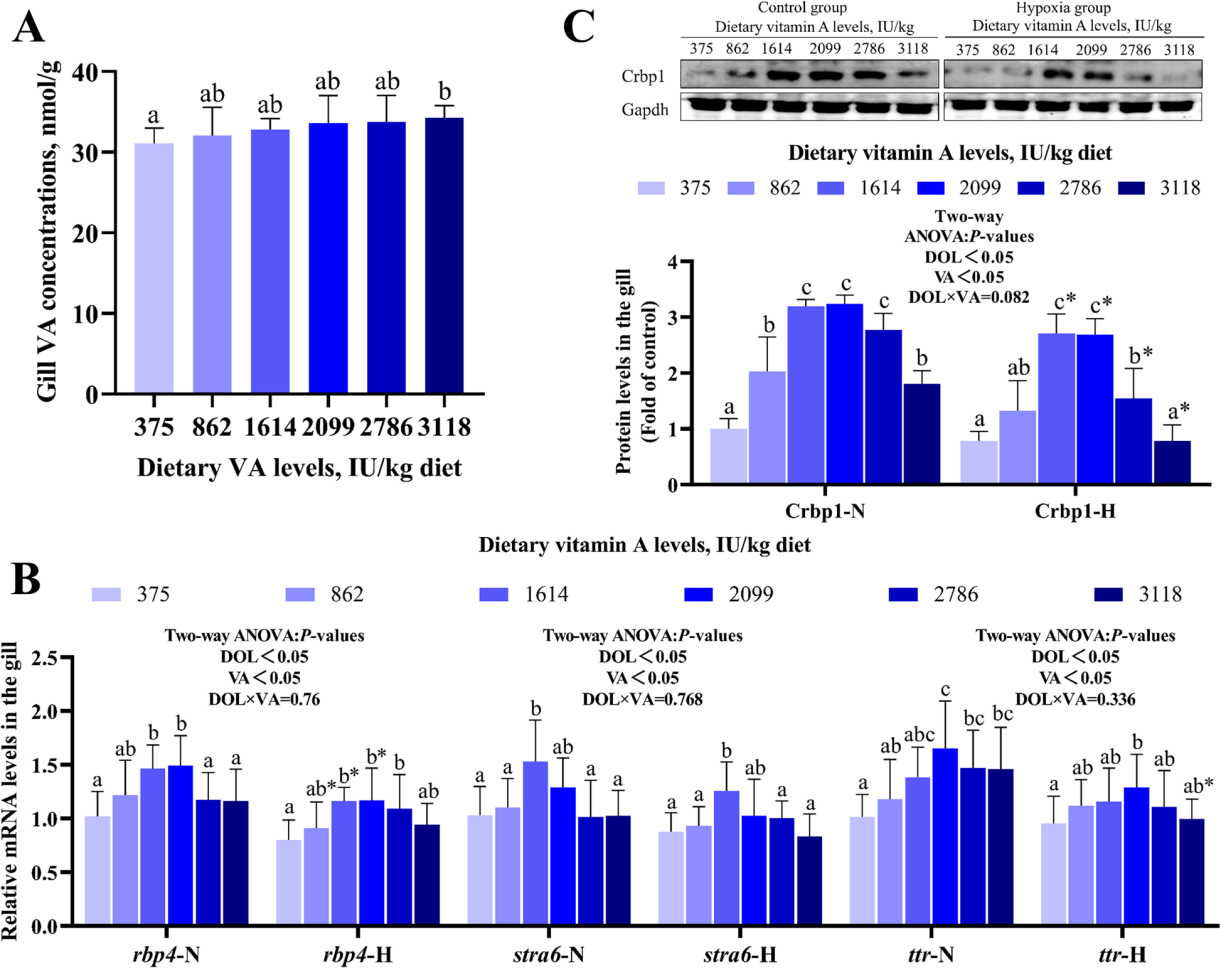
Effects of vitamin A on gill VA transporters in grass carp following hypoxic stress. **A** VA concentration in gills (*n* = 8). **B** The mRNA levels of VA transporter-related genes in gills (*n* = 8). **C** Protein abundance of VA transporters in gills (*n* = 4). Values are means ± standard deviation. Different letters indicate significant differences between VA treatments at the same dissolved oxygen level (*P* < 0.05). “*” indicates significant differences between normoxic and hypoxic groups at the same VA level (*P* < 0.05)

As shown in Fig. 2A, gill VA concentration reached its maximum at a dietary VA level of 3,118 IU/kg. Under both normoxic and hypoxic conditions (Fig. 2B), mRNA expression of retinol binding protein 4 (*rbp4*) was significantly elevated within the 1,614–2,099 IU/kg VA range compared to the 375 IU/kg group; the expression of stimulated by retinoic acid 6 (*stra6*) and thyroxine transport protein (*ttr*) peaked at 1,614 and 2,099 IU/kg VA, respectively; similarly, the protein abundance of Crbp1 increased significantly in both oxygen conditions within the same VA range (1,614–2,099 IU/kg) (*P* < 0.05). However, hypoxic groups exhibited significantly lower levels of *rbp4*, *ttr*, and Crbp1 compared to their normoxic counterparts (*P* < 0.05). Two-way ANOVA indicated that the expression of *rbp4*, *stra6*, *ttr*, and Crbp1 was not significantly influenced by the interaction between DO and VA (*P* > 0.05).

### Gill morphology and oxidative damage

**Fig. 3:**
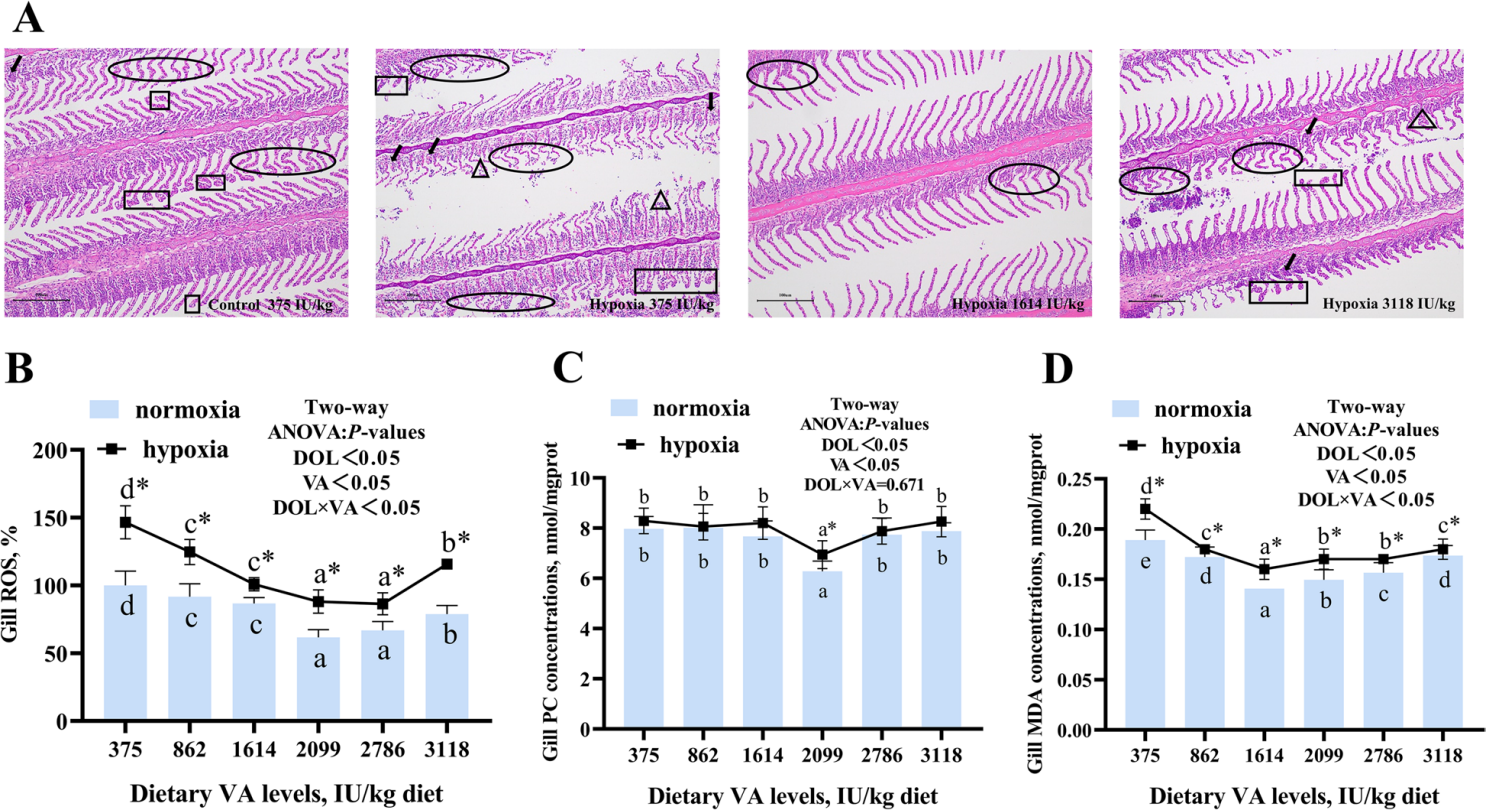
Effects of vitamin A on gill histology and oxidative stress in grass carp following hypoxic stress. **A** Representative photomicrographs of gill sections (H&E staining, 200 × magnification). Triangles: epithelial bulges; circles: gill plates curled; squares: epithelial swellings; arrows: hyperplasia. **B** Reactive oxygen species (ROS) levels in gills (*n* = 8). **C** Protein carbonyl (PC) content in gills (*n* = 8). **D** Malondialdehyde (MDA) concentration in gills (*n* = 8). Values are means ± standard deviation. Different letters indicate significant differences between VA treatments at the same dissolved oxygen level (*P* < 0.05). “*” indicates significant differences between normoxic and hypoxic groups at the same VA level (*P* < 0.05)

As shown in Fig. 3A, gill tissue damage—including epithelial bulging, swelling, lamellar curling, and hyperplasia—was more severe in hypoxic groups fed 375 and 3,118 IU/kg VA compared to the normoxic group at 375 IU/kg. These pathological changes were markedly alleviated in hypoxic fish fed 1,614 IU/kg VA. Under both normoxic and hypoxic conditions, ROS levels were significantly reduced within the VA range of 864–3,118 IU/kg compared to the 375 IU/kg group. PC and MDA levels reached their minimum at VA levels of 2,099 and 1,614 IU/kg, respectively (Fig. 3B–D; *P* < 0.05). Hypoxic groups exhibited significantly higher levels of ROS, PC, and MDA relative to their normoxic counterparts at the same VA levels (*P* < 0.05). Two-way ANOVA indicated that ROS and MDA levels were significantly influenced by DO, VA, and their interaction (*P* < 0.05).

### Gill mitochondrial stress

**Fig. 4:**
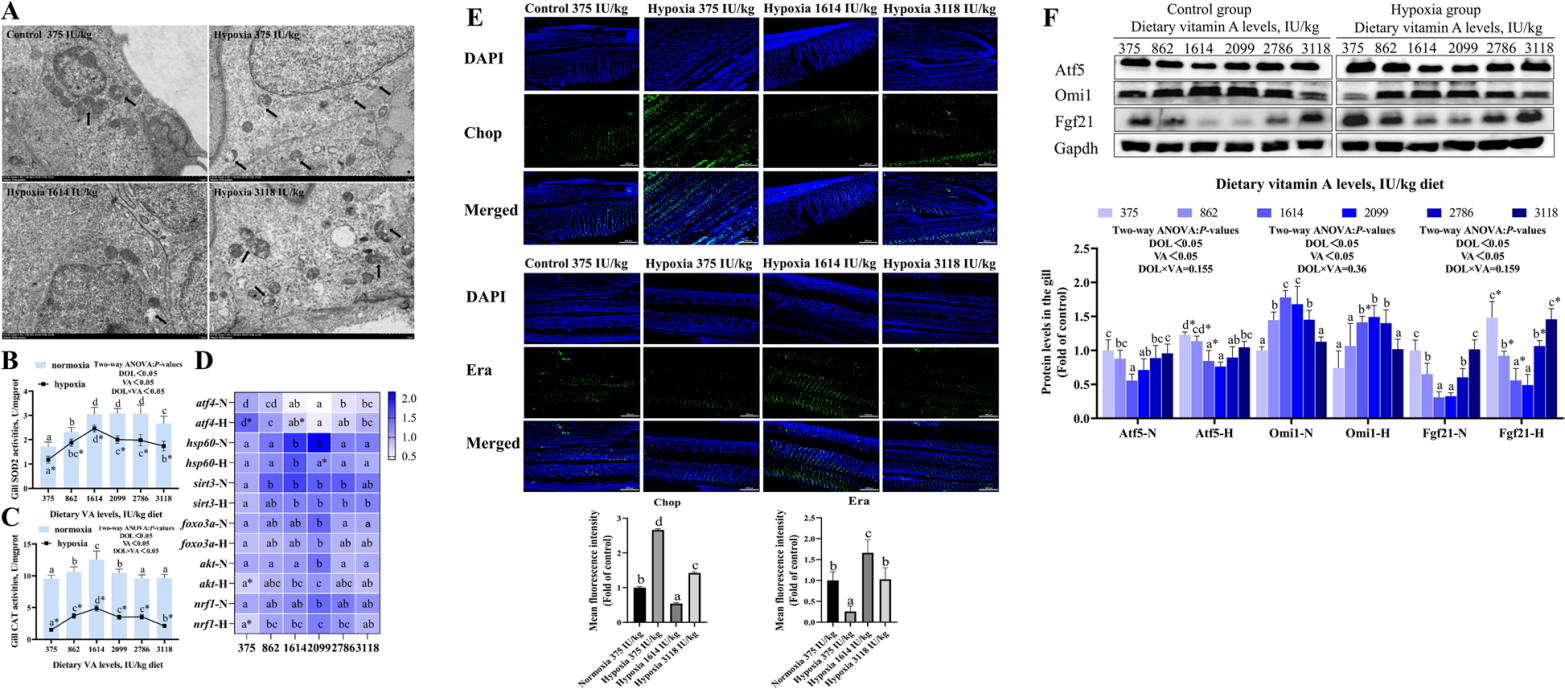
Effects of vitamin A on mitochondrial stress in the gills of grass carp following hypoxic stress. **A** Representative transmission electron micrographs of gill tissue (10,000 × magnification). Arrows: damaged mitochondria. **B** Superoxide dismutase 2 (SOD2) activity in gills (*n* = 8). **C** Catalase (CAT) activity in gills (*n* = 8). **D** The mRNA levels of mitochondrial stress-related genes in gills (*n* = 8). **E** Immunofluorescence staining of mitochondrial stress-related markers. **F** Protein levels of mitochondrial stress-related markers in gills (*n* = 4). Values are means ± standard deviation. Different letters indicate significant differences between VA treatments at the same dissolved oxygen level (*P* < 0.05). “*” indicates significant differences between normoxic and hypoxic groups at the same VA level (*P* < 0.05)

Mitochondrial ultrastructure in grass carp gill cells was examined by transmission electron microscopy (Fig. 4A). Compared to normoxic groups, hypoxic groups fed 375 or 3,118 IU/kg VA exhibited pronounced mitochondrial damage, including fragmentation of mitochondria and loss of cristae. These morphological abnormalities were markedly alleviated in hypoxic fish fed 1,614 IU/kg VA.

As shown in Fig. 4B and C, the activities of SOD2 and CAT exhibited a quadratic response to increasing dietary VA levels, peaking at 1,614 IU/kg VA (*P* < 0.05). Under hypoxia, SOD2 and CAT activities were significantly reduced at several VA concentrations compared to normoxic conditions. Two-way ANOVA indicated that both DO and VA, as well as their interaction, significantly influenced SOD2 and CAT activities (*P* < 0.05).

In both normoxic and hypoxic groups, the mRNA expression of heat shock protein 60 (*hsp60*), sirtuin3 (*sirt3*), forkhead box O3 (*foxo3a*), *akt* and *nrf1* followed a quadratic pattern, initially increasing and then decreasing with rising dietary VA levels, a trend opposite to that of *atf4* (Fig. 4D; *P* < 0.05). Specifically, compared to the 375 IU/kg VA group, *hsp60* expression was significantly higher at 1,614 IU/kg VA; *sirt3* was elevated within the range of 1,614–2,786 IU/kg VA; and *foxo3a*, *akt*, and *nrf1* reached their peak expression at 2,099 IU/kg VA (*P* < 0.05). Under hypoxic conditions, mRNA levels of *hsp60*, *akt*, and *nrf1* were significantly reduced at several VA concentrations, while *atf4* expression was increased, compared to normoxic groups. Furthermore, two-way ANOVA revealed that the interaction between DO and VA significantly influenced *hsp60* mRNA levels (*P* < 0.05).

Compared to the normoxic group, the hypoxic group showed a significant reduction in Chop fluorescence intensity at VA levels of 1,614 and 3,118 IU/kg (Fig. 4E; *P* < 0.05). Conversely, the fluorescence intensity of Erα was significantly increased under hypoxic conditions at the 1,614 IU/kg VA level (*P* < 0.05).

In Fig. 4F, compared to the 375 IU/kg VA group, protein levels of activating transcription factor 5 (Atf5) and fibroblast growth factor 21 (Fgf21) were significantly reduced within the 2,099–2,786 IU/kg VA range under both normoxic and hypoxic conditions, a trend opposite to that observed for the serine protease Omi1 (*P* < 0.05). Hypoxic exposure led to significantly higher Atf5 and Fgf21 levels, but lower Omi1 levels, at several VA concentrations compared to normoxic controls (*P* < 0.05). However, two-way ANOVA indicated that the expression of Atf5, Fgf21, and Omi1 was not significantly influenced by the interaction between DO and VA (*P* > 0.05).

### Gill mitochondrial quality control

**Fig. 5:**
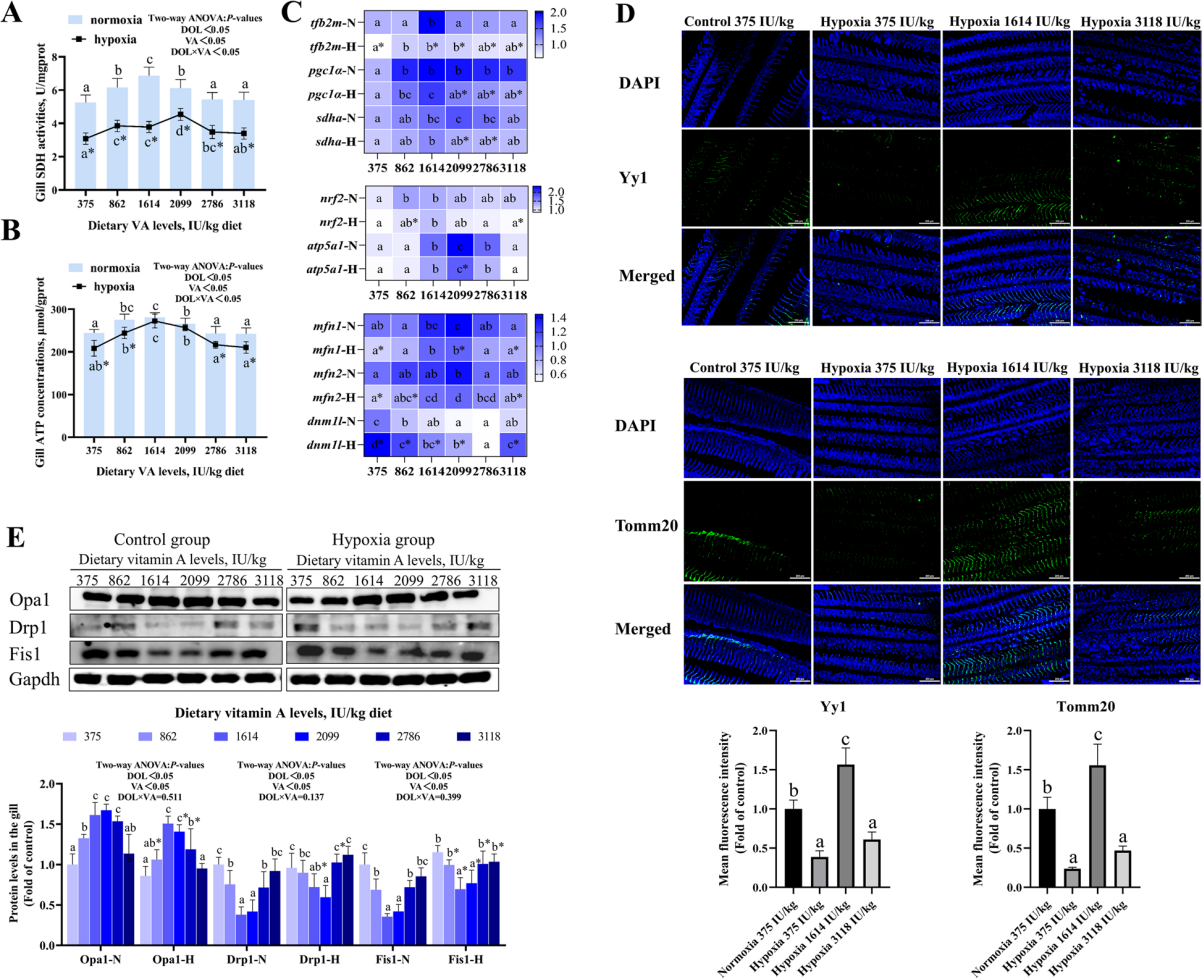
Effects of vitamin A on mitochondrial quality control (MQC) in the gills of grass carp following hypoxic stress. **A** Succinate dehydrogenase (SDH) activity in gill tissue (*n* = 8). **B** Adenosine triphosphate (ATP) concentration in gills (*n* = 8). **C** The mRNA expression of MQC-related genes in gills (*n* = 8). **D** Immunofluorescence analysis of MQC-associated proteins. **E** Protein levels of MQC-related markers (*n* = 4). Values are means ± standard deviation. Different letters indicate significant differences between VA treatments at the same dissolved oxygen level (*P* < 0.05). “*” indicates significant differences between normoxic and hypoxic groups at the same VA level (*P* < 0.05)

Compared to the 375 IU/kg group, SDH levels were significantly higher in the range of 864–2,099 IU/kg VA levels, and ATP content reached a maximum at 1,614 IU/kg VA level in both normoxic and hypoxic groups (Fig. 5A and B; *P* < 0.05). SDH and ATP levels of hypoxic groups reduced significantly compared with normoxic groups (*P* < 0.05). Furthermore, a significant interactive effect between DO and VA was observed on both SDH and ATP levels (*P* < 0.05).

In both normoxic and hypoxic groups, mRNA levels of transcription factor B2, mitochondrial (*tfb2m*), *pgc1α*, succinate dehydrogenase complex subunit a (*sdha*), nuclear respiratory factor 2 (*nrf2*), and ATP synthase α subunit (*atp5a1*) were significantly increased at a VA concentration of 1,614 IU/kg; mRNA levels of *mfn1/2* were significantly increased at 2,099 IU/kg VA; the mRNA level of dynamin 1 like (*dnm1l*) was significantly reduced across VA levels ranging from 864 to 3,118 IU/kg (Fig. 5C; *P* < 0.05). Compared with normoxic groups, excluding *dnm1l*, expression of above genes in the hypoxic group significantly reduced at some VA levels. Notably, only *tfb2m* and *dnm1l* levels were significantly impacted by the interaction between DO and VA (*P* < 0.05).

In hypoxic groups, the fluorescence intensity of Yy1 and Tomm20 was significantly higher at the 1,614 IU/kg VA level compared to normoxic groups (Fig. 5D; *P* < 0.05).

As shown in Fig. 5E, under both normoxic and hypoxic conditions, protein levels of Opa1 were increased within the VA range of 1,614–2,786 IU/kg compared to the 375 IU/kg group; In contrast, protein levels of Drp1 and Fis1 were significantly reduced at VA concentrations between 1,614 and 2,099 IU/kg (*P* < 0.05). Under hypoxia, Opa1 levels were decreased, while Drp1 and Fis1 levels were elevated relative to normoxic groups at corresponding VA concentrations (*P* < 0.05). However, Opa1, Drp1, and Fis1 were not affected by the interaction between DO and VA (*P* > 0.05).

### Gill mitophagy

**Fig. 6:**
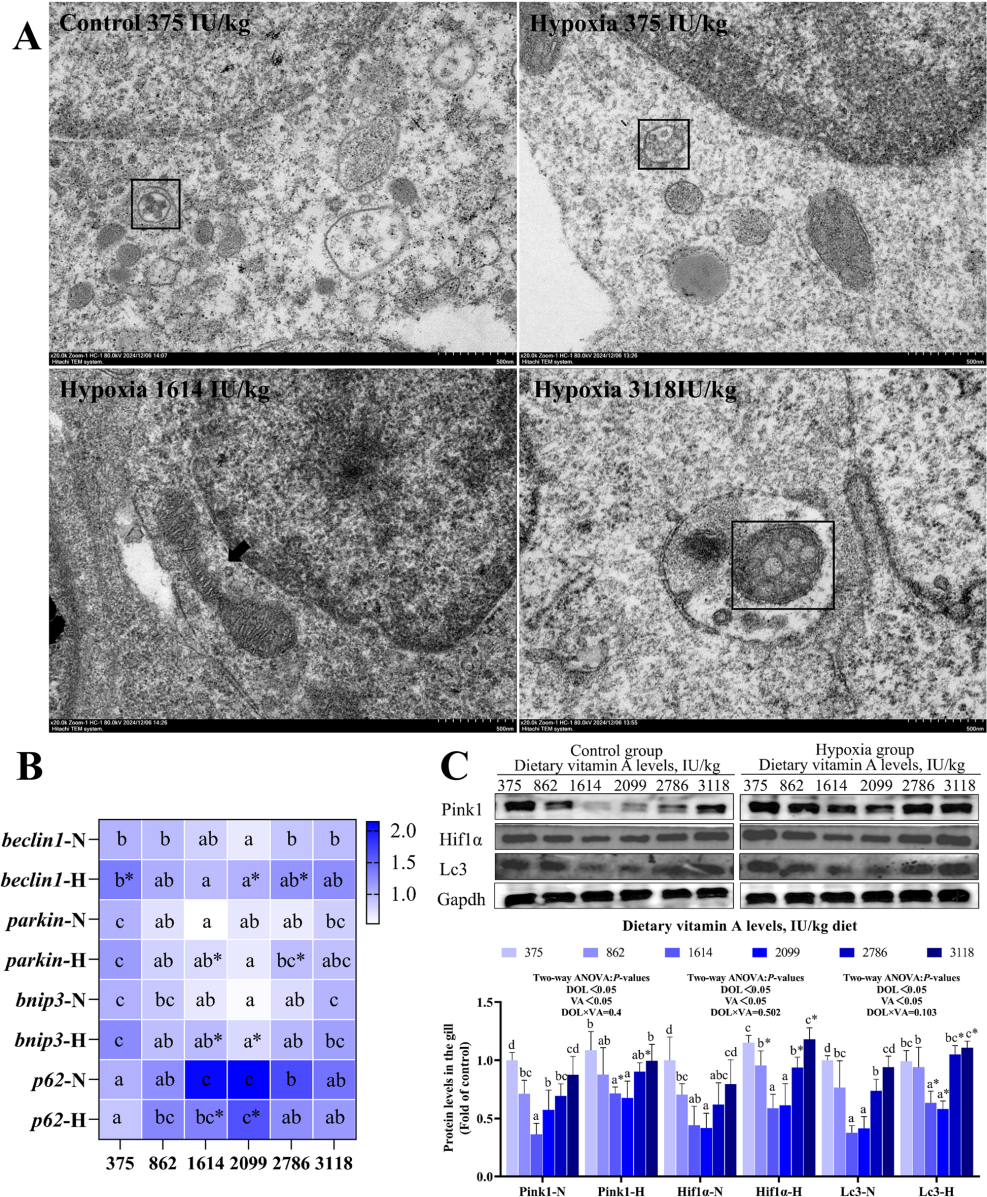
Effects of vitamin A on mitophagy in the gills of grass carp following hypoxic stress. **A** Representative transmission electron micrographs of gill tissue (20,000 × magnification). Square: autophagosome; arrow: damaged mitochondria. **B** The mRNA levels of mitophagy-related genes in gills (*n* = 8). **C** Protein expression of mitophagy-related markers (*n* = 4). Values are means ± standard deviation. Different letters indicate significant differences between VA treatments at the same dissolved oxygen level (*P* < 0.05). “*” indicates significant differences between normoxic and hypoxic groups at the same VA level (*P* < 0.05)

As shown in Fig. 6A, autophagosomes were observed in both the normoxic group (375 IU/kg) and the hypoxic groups (375 and 3,118 IU/kg). Slightly damaged mitochondria were detected in the hypoxic 1,614 IU/kg group; however, no mitophagy was observed.

In Fig. 6B, in both normoxic and hypoxic groups, compared to the 375 IU/kg group, mRNA levels of BCL-2-interacting myosin-like coiled-coil protein (*beclin1*), *parkin* and *bnip3* were significantly lower at 2,099 IU/kg VA level; while autophagy receptor protein 62 (*p62*) was significantly higher in the range of 1,614–2,099 IU/Kg VA levels (*P* < 0.05). Hypoxic groups exhibited higher expression of *beclin1*, *parkin*, and *bnip3*, but lower levels of *p62* compared to normoxic groups at the same VA concentrations (*P* < 0.05). Additionally, *p62* expression was significantly influenced by the interaction between DO and VA (*P* < 0.05).

Compared to the 375 IU/kg VA group, protein levels of Pink, Hif1α, and microtubule-associated protein 1 light chain 3 (Lc3) were reduced in 1,614–2,099 IU/Kg VA levels under both normoxic and hypoxic groups (Fig. 6C; *P* < 0.05). Relative to normoxic groups, hypoxic groups exhibited increased protein levels of Pink, Hif1α and Lc3 (*P* < 0.05). However, the expression of these proteins was not significantly affected by the interaction between DO and VA (*P* > 0.05).

### Regression analysis

**Fig. 7:**
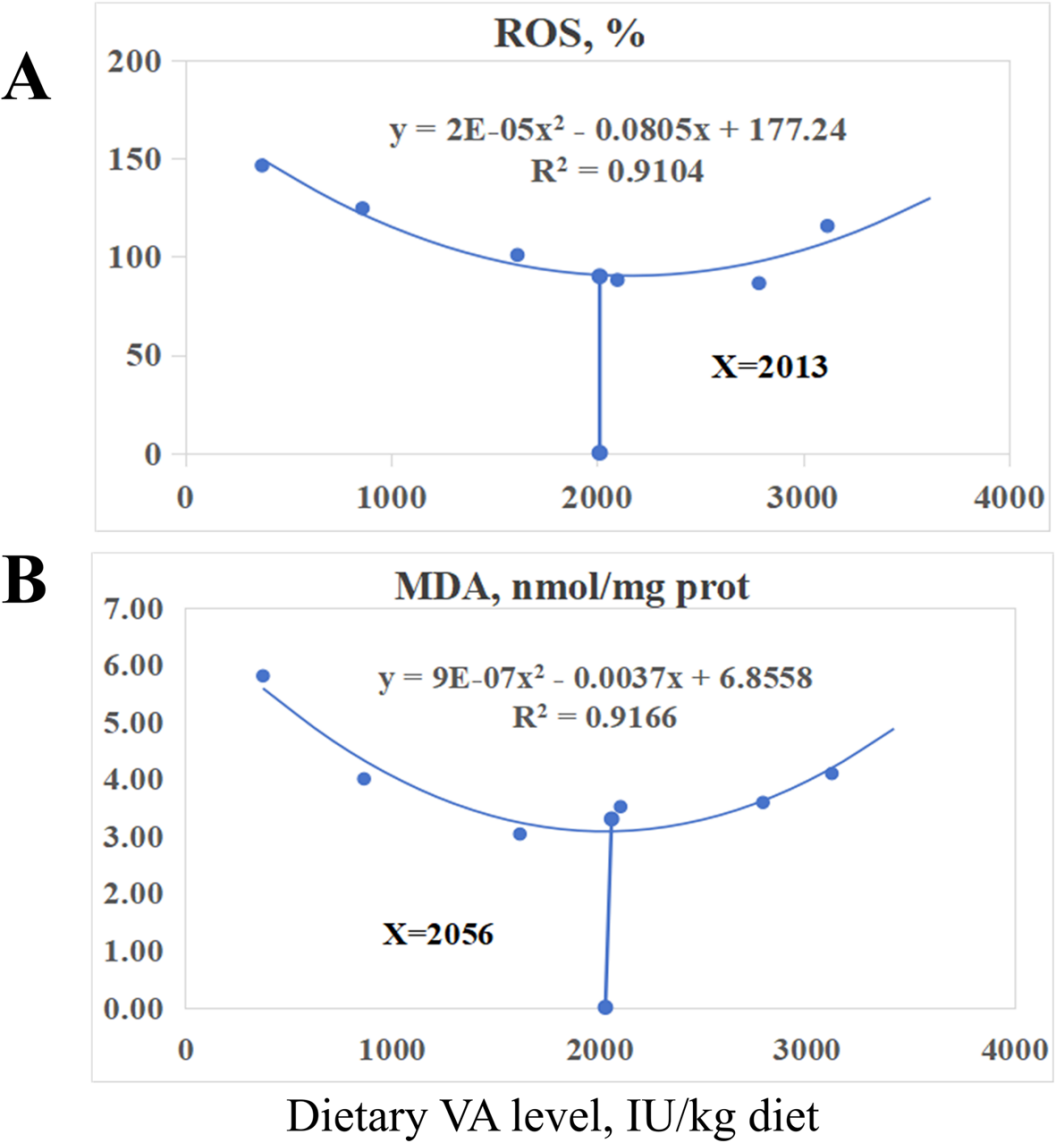
Quadratic regression analyses of (**A**) reactive oxygen species (ROS) content, and (**B**) malondialdehyde (MDA) content in relation to dietary vitamin A levels in adult grass carp

Based on quadratic regression analysis of ROS and MDA from the hypoxic group (Fig. 7), the estimated VA requirements for adult grass carp were determined to be 2,013 and 2,056 IU/kg diet, respectively.

## Discussion

Hypoxia is a common challenge in grass carp aquaculture. As the primary respiratory organ in fish, gills are particularly susceptible to damage under such stress [4]. Here, we present the first report on the influence and potential mechanisms of VA in mitigating mitochondrial stress and regulating MQC in the gills of grass carp under hypoxic conditions.

### VA promoted the transport of VA to the gills

The accumulation of VA in tissues is a widely used indicator for determining VA requirements in aquatic animals [30]. We observed that a dietary level of 3,118 IU/kg VA increased the VA content in gills of grass carp compared to lower VA levels, indicating enhanced VA deposition through exogenous VA supplementation. Crbp1 facilitates the transport of VA into organelles [31]. The cell surface Rbp4 receptor is identified as Stra6 [32]. Rbp4 binds VA and transports it to various organs via the bloodstream [33]. Meanwhile, the VA-Rbp4 complex stabilizes by forming a complex with Ttr [34]. Here, VA upregulated the protein expression of Crbp1 and the mRNA levels of *stra6, rbp4* and *ttr*, but the interaction between VA and DO had no effect on these indicators, indicating that the transport mode of VA did not change under hypoxia.

### VA attenuated gill damage induced by hypoxia in fish

Elevated serum LD levels are often indicative of mitochondrial dysfunction [35]. Previous study has also reported a correlation between LDH activity and intracellular oxygen partial pressure, suggesting its potential use as an indicator of hypoxic stress [36]. In the present study, dietary VA supplementation within the range of 862–2,099 IU/kg reduced the elevated serum LD content and LDH activity under hypoxic conditions, but the interaction between VA and DO had no effect on LD, indicating that VA alleviated the hypoxic state of fish mainly by changing LDH activity.

As the primary respiratory organ in fish, gills play a crucial role in responding to hypoxic stress. Exposure to hypoxia can cause structural damage to gill tissues [3]. In our study, dietary supplementation of VA at 1,614 IU/kg reduced hypoxic-induced morphological alterations, including epithelial bulging, swelling, lamellar curling, and hyperplasia, suggesting that VA helped maintain gill structural integrity and supports adaptation to hypoxia.

Hypoxia disrupts the electron transport pathway, leading to accumulation of ROS, which in turn induces oxidative damage to lipids and proteins [37]. Consistent with this, we observed elevated levels of ROS, PC, and MDA in the gills of hypoxic groups. VA supplementation (up to 2,099 IU/kg) significantly reduced these markers, and a significant interaction with DO was observed, indicating that VA enhanced antioxidant protection under hypoxic conditions. This antioxidant capacity may be attributed to the chemical structure of VA: its conjugated polyene chain enables it to quench singlet oxygen, neutralize thioredoxin radicals and stabilize peroxyl radicals [38]. Elevated oxidative stress is known to induce mitochondrial dysfunction [39], which can promote a proteotoxic environment and exacerbate mitochondrial stress [12]. Given these relationships, we further investigated the influence of VA on mitochondrial stress response pathways.

### VA alleviated mitochondrial stress induced by hypoxia in fish

Mitochondria respond to stress by coordinating metabolic pathways that maintain cellular energy homeostasis and redox balance [40, 41]. Mitochondrial cristae, the primary site of oxidative phosphorylation, play an essential role in cellular energy production [42]. Electron microscopy revealed that dietary VA supplementation at 1,614 IU/kg attenuated hypoxia-induced mitochondrial fragmentation and cristae disruption. These findings suggested that VA enhanced hypoxic adaptability in fish by preserving mitochondrial integrity and ultrastructure.

Fgf21, a soluble factor released by cells under mitochondrial stress, serves as a key biomarker of such conditions [43]. Under hypoxic stress, VA significantly downregulated the protein level of Fgf21, but the interaction effect of VA and DO had no significant effect on it, indicating that VA played a regulatory role independent of hypoxia. UPR^mt^ involves changes in the localization and expression of Chop, Atf4, and Atf5, leading to the induction of mitochondrial chaperones such as HSP60 to enhance protein-folding capacity within mitochondria [12]. In this study, hypoxia increased Chop immunofluorescence intensity, upregulated *atf4* gene expression and Atf5 protein levels, and downregulated the mRNA of *hsp60*. VA supplementation counteracted these changes by reducing Chop, *atf4*, and Atf5 levels while increasing *hsp60* expression. The *hsp60* levels were significantly affected by the interaction between VA and DO. These results suggest that VA enhances the resistance of grass carp to hypoxic stress by modulating the canonical UPR^mt^ pathway, thereby restoring mitochondrial proteostasis and reducing stress-related signaling.

As a part of the UPR^mt^ sirtuin axis, Sirt3 activation promotes the deacetylation of Foxo3a, resulting in the induction of antioxidant enzymes such as SOD2 and CAT [12]. In this study, hypoxia reduced the activities of SOD2 and CAT and downregulated the mRNA levels of *sirt3* and *foxo3a*. VA supplementation increased the expression and activity of SOD2, CAT, *sirt3*, and *foxo3a*, the interaction between VA and DO significantly influenced the levels of SOD2 and CAT, demonstrating that fish fed higher levels of VA (2,099 IU/kg) exhibited enhanced antioxidant capacity, thereby alleviating hypoxic stress.

The transcriptional response of canonical UPR^mt^ axis that maintains mitochondrial health is probably highly complementary to the antioxidant action of the UPR^mt^ sirtuin axis [12]. In addition, misfolded proteins in the membrane gap trigger the UPR^IMS^/Erα axis, which acts through Akt and ROS-dependent phosphorylation of Erα, resulting in induction of Nrf1, increased protease levels and activity (e.g., mitochondrial serine protease Omi/HtrA2), and increased protein quality control [12]. In this study, hypoxia downregulated the mRNA levels of *akt* and *nrf1*, reduced Omi1 protein expression, and decreased Erα immunofluorescence intensity. VA supplementation upregulated the expression of *akt, nrf1,* Omi1*,* and Erα, these results indicated that VA enhanced the resistance to mitochondrial stress and alleviated hypoxic stress by regulating the UPR^IMS^/Erα axis.

In conclusion, VA (1,614–2,099 IU/kg) suppressed mitochondrial stress, which was associated with inhibition of the canonical UPR^mt^ axis and promotion of the UPR^mt^ sirtuin axis and UPR^IMS^/Erα axis. Given that mitochondrial stress is known to reprogram mitochondrial biogenesis and metabolism [14], we studied the impact of VA on MQC under hypoxic stress.

### VA alleviated abnormal mitochondrial quality control induced by hypoxia in fish

Mitochondrial function is regulated by the mitochondrial quality control system, which maintains mitochondrial morphology, quantity, and quality through processes such as fission, fusion, and biogenesis [44]. Key regulators include Tfb2m, a classical transcription factor for mitochondrial DNA transcription and replication [45], Pgc1α, which stimulates mitochondrial biogenesis [46], Sdha, a marker of mitochondrial content [47], Tomm20, an indicator of mitochondrial quality [48]. In our study, hypoxia decreased SDH activity, downregulated the mRNA levels of *tfb2m*, *pgc1α* and *sdha*, and reduced Tomm20 immunofluorescence intensity, VA increased SDH, *tfb2m*, *pgc1α, sdha* and Tomm20 levels in grass carp gills. The interaction between VA and DO had a significant impact on SDH, ATP, and *tfb2m*. These results demonstrated that VA enhanced mitochondrial content and function, thereby improving the anti-stress capacity of fish under hypoxic conditions.

Mitochondria serve as essential cellular energy generators. The expression of respiratory genes is regulated by transcription factors such as Nrf2 and Yy1 [49]. Additionally, Atp5a1 encodes a critical subunit of mitochondrial ATP synthase [50]. In the study, hypoxia reduced ATP content, downregulated the mRNA levels of *nrf2* and *atp5a1*, and decreased Yy1 immunofluorescence intensity, VA increased ATP, *nrf2*, *atp5a1*, and Yy1 levels in the gills. These results indicated that VA enhanced respiratory capacity and promoted mitochondrial energy production under hypoxic conditions, thereby improving the ability of fish to adapt to hypoxic environments.

Mitochondrial homeostasis is mainly adjusted by mitochondrial fission and fusion (mitochondrial dynamics) and biogenesis, and external stresses can disrupt this homeostasis [51]. Mfn1 and Mfn2 interact to facilitate the fusing of mitochondrial outer membranes, while OPA1 is responsible for fusion of the inner membranes [52]. Conversely, Dnm1L and Fis1 are involved in mitochondrial fission [53]. Drp1 interacts with receptor Fis1 to complete mitochondrial fission [54]. In our study, hypoxia downregulated the mRNA levels of *mfn1* and *mfn2* and reduced Opa1 protein expression, while upregulating *dnm1l* gene expression and increasing Drp1 and Fis1 protein levels. VA upregulated *mfn1, mfn2* and Opa1 levels, and downregulated *dnm1l*, Drp1 and Fis1 levels. The interaction between VA and DO only had a significant impact on *dnm1l*. These results demonstrated that VA helped maintain mitochondrial dynamic balance under hypoxic conditions by modulating key fusion and fission factors, thereby enhancing mitochondrial stability and function.

In short, VA (1,614–2,786 IU/kg) supported mitochondrial biogenesis and function, and maintained mitochondrial dynamics homeostasis to control mitochondrial mass. Since mitochondrial quantity is determined by the interplay between biogenesis, dynamics, and mitophagy [55]. We further investigated the influence of VA on mitophagy.

### VA alleviated mitophagy induced by hypoxia in fish

Mitophagy is the process through which damaged mitochondria are selectively engulfed and degraded to maintain the integrity and homeostasis of the mitochondrial network [56]. Transmission electron microscopy showed a reduced number of autophagosomes in the gills of the 1,614 IU/kg VA group, indicating that VA mitigated hypoxia-induced excessive mitophagy. Under stress, damaged mitochondria may be transported via microtubules to lysosomes for degradation [57]. Bnip3, a downstream target of Hif1α, binds directly to Lc3 and promotes mitophagy initiation [58]. In this study, hypoxia upregulated the expression of *beclin1*, *parkin*, and *bnip3*, downregulated *p62* mRNA levels, and increased the protein levels of Pink, Hif1α, and Lc3. VA supplementation reversed these changes by downregulating *beclin1*, *parkin*, *bnip3*, Pink, Hif1α, and Lc3, while upregulating *p62* expression, the interaction between VA and DO had a significant impact on *p62*, demonstrating that VA inhibited the overactivation of mitophagy under hypoxia, thereby contributing to the maintenance of mitochondrial homeostasis. This might be closely linked to the Pink1/Parkin and Hif1a-Bnip3 pathway.

### The VA requirements for grass carp to resist hypoxic stress

According to the quadratic regression analysis of ROS and MDA from the hypoxic group (Fig. 7), the VA requirements for adult grass carp were determined to be 2,013, and 2,056 IU/kg diet, respectively. This is slightly higher than that we discovered in our study regarding the growth requirement (1,921 IU/kg diet; unpublished data), indicating that oxidative stress caused by hypoxia led to tissue oxidative damage, and cells required higher levels of VA to repair or protect damaged tissues.

## Conclusion

The potential mechanisms by which VA maintained gill health in fish could be summarized as follows: First, optimal VA levels (1,614–2,099 IU/kg) suppressed mitochondrial stress, which might be associated with the inhibition of the canonical UPR^mt^ axis and the promotion of the UPR^mt^ sirtuin axis and UPR^IMS^/Erα axis. Second, optimal VA levels (1,614–2,786 IU/kg) supported mitochondrial biogenesis and function, and maintained mitochondrial dynamics homeostasis to control mitochondrial mass. Third, the optimal VA levels (1,614–2,099 IU/kg) inhibited excessive mitophagy, and its mechanism might be connected to the suppression of Pink1/Parkin dependent pathway and Hif1a-Bnip3 pathway.

## Supplementary Information

Additional file 1. Table S1. Components and nutrition levels of the trial feeds. Table S2. Biochemical index determination kit number. Table S3. The information of antibodies (Western blot and immunofluorescence). Table S4. Real-time PCR primer sequences. 

Additional file 2: The original gel and blot images.

## Data Availability

Data will be made available from the corresponding author on request.

## Abbreviations

*atf4*: Activating transcription factor 4
Atf5: Activating transcription factor 5
*atp5al*: ATP synthase α subunit
*becline1*: BCL-2-interacting myosin-like coiled-coil protein
*bnip3*: Bcl-2 adenovirus E1 B 19 kDa interacting protein 3
Chop: C/EBP homologous protein
Crbp1: Cellular retinol-binding protein 1
*dnm1l*: Dynamin 1 like
Drpl: Dynamin-related protein 1
Era: Estrogen receptor a
Fgf21: Fibrolast growth factor 21
Fis1: Mitochondrial fission 1
*foxo3a*: Forkhead box O3
Hif1a: Hypoxia-inducible factor 1a
*hsp60*: Heat shock protein 60
Lc3: Microtubule-associated protein 1 light chain 3
*mfn1/2*: Mitochondrial fusion protein 1/2
*nrf1/2*: Nuclear respiratory factor 1/2
Opa1: Optic atrophy protein
*p62*: Autophagy receptor protein 62
*pgc1a*: Proliferator-activated receptor gamma coactivator 1a
Pink1: PTEN-induced putative kinase 1
*rbp4*: Retinol binding protein 4
*sdha*: Succinate dehydrogenase complex subunit a
*sirt3*: Sirtuin 3
*stra6*: Stimulated by retinoic acid 6
*tfb2m*: Transcription factor B2, mitochondrial
Tomma: Translocase of outer mitochondrial membrane 20
ttr: Thyroxin transport protein
