# Supplementary material for: Exploring new benefits of vitamin A: alleviating hypoxia-induced mitochondrial stress and mitophagy in the gills of adult grass carp (Ctenopharyngodon idellus)
Source: J Anim Sci Biotechnol. 2025 Dec 16;16:172. doi: 10.1186/s40104-025-01309-3 (PMC12706901; doi:10.1186/s40104-025-01309-3)
Supplement: Supplementary file 1 — Additional file 1. Table S1. Components and nutrition levels of the trial feeds. Table S2. Biochemical index determination kit number. Table S3. The information of antibodies (Western blot and immunofluorescence). Table S4. Real-time PCR primer sequences. [file 40104_2025_1309_MOESM1_ESM.docx]

Table S1 Components and nutrition levels of the trial feeds.

| **Constituents** | **Content (%)** | **Nutrients** | **Content (%)** |
| --- | --- | --- | --- |
| Fish meal | 8.00 | Crude protein^e^ | 26.00 |
| Soybean protein concentrate | 19.00 | Crude lipid^e^ | 4.01 |
| Cottonseed protein concentrate | 13.14 | n-3^f^ | 1.129 |
| L-Threonine (97.5%) | 0.16 | n-6 ^f^ | 1.10 |
| L-Tryptophan (98%) | 0.23 | Available phosphorus ^f^ | 0.40 |
| flaxseed oil | 1.55 |  |  |
| Soybean oil | 1.52 |  |  |
| α-starch | 20.00 |  |  |
| Corn starch | 25.085 |  |  |
| Butylated hydroxyanisole | 0.015 |  |  |
| Ca(H_2_PO_4_)_2_ | 1.30 |  |  |
| Microcrystalline cellulose | 5.00 |  |  |
| Vitamin premix (Vitamin A free)^a^ | 1.00 |  |  |
| Mineral premix^b^ | 2.00 |  |  |
| Choline chloride premix^c^ | 1.00 |  |  |
| Vitamin A premix^d^ | 1.00 |  |  |
| Total | 100.00 |  |  |

^a^ Vitamin premix per kilogram (g/kg): vitamin D_3_ (500,000 IU/g), 0.19 g; DL-a-tocopherol acetate (50%), 25.50 g; vitamin K_3_ (50%), 0.38 g; riboflavin (80%), 0.73 g; vitamin B_6_ (98%), 0.55 g; calcium-D-pantothenate (93.1%), 4.42 g; niacin (99%), 3.33 g; meso-inositol (96.5%), 28.67 g; vitamin B_12_ (1%), 0.94 g; D-biotin (2%), 1.05 g; folic acid (95%), 0.17 g; vitamin C acetate (95%), 9.77 g. All components were supplemented to 1 kg with corn starch.

^b^ Mineral premix per kilogram (g/kg): MnSO_4_•H_2_O (31.8% Mn), 2.66 g; MgSO_4_•H_2_O (15.0% Mg), 256.79 g; FeSO_4_•H_2_O (30.0% Fe), 12.61 g; ZnSO_4_•H_2_O (34.5% Zn), 8.87 g; CuSO_4_•5H_2_O (25.0% Cu), 0.95 g; Ca (IO_3_)_2_ (3.2% I), 1.56 g; yeast selenium (0.2% Se), 13.65 g. All components were supplemented to 1 kg with corn starch.

^c^ Choline chloride premix: choline chloride (50%), 261.95 g; the remainder was supplemented to 1 kg with corn starch.

^d^ Vitamin A premix per kilogram (IU/kg): premixes were incorporated into the base feed to give experimental feeds with distinct VA levels. The measured VA levels of feeds were 375, 862, 1614, 2099, 2786, and 3118 IU/kg diet.

^e^ Values of crude protein and lipid were tested.

^f^ Available phosphorus, n-3, and n-6 levels were assessed based on **National Research Council** (NRC) data (Resources, 2011).

Table S2 Biochemical index determination kit number

| Kits | Product Number | Manufacturers |
| --- | --- | --- |
| LD | A019-2-1 | Nanjing Jiancheng Bioengineering Institute |
| LDH | A020-2-2 | Nanjing Jiancheng Bioengineering Institute |
| SOD2 | A001-3-2 | Nanjing Jiancheng Bioengineering Institute |
| CAT | A007-1-1 | Nanjing Jiancheng Bioengineering Institute |
| SDH | A022-1-1 | Nanjing Jiancheng Bioengineering Institute |
| ROS | E004-1-1 | Nanjing Jiancheng Bioengineering Institute |
| PC | A087-1-2 | Nanjing Jiancheng Bioengineering Institute |
| MDA | A003-1-2 | Nanjing Jiancheng Bioengineering Institute |
| ATP | A095-1-1 | Nanjing Jiancheng Bioengineering Institute |
| VA | DM-TY46136 | Duma Biology |

Table S3 The information of antibodies (Western blot and immunofluorescence)

| **Antibodies** | **Host** | **Product number** | **Product source** | **Dilution** |
| --- | --- | --- | --- | --- |
| Chop | Rabbit | A21902 | Abclonal, China | 1:200 |
| Fgf21 | Rabbit | ET1704-04 | Huabio, China | 1:4000 |
| Crbp1 | Rabbit | ER1906-63 | Huabio, China | 1:1500 |
| Opa1 | Rabbit | HA722673 | Huabio, China | 1：4000 |
| Drp1 | Rabbit | A16661 | Abclonal, China | 1：4000 |
| Atf5 | Rabbit | ET1612-38 | Huabio, China | 1：2000 |
| Lc3 | Rabbit | ET1701-65 | Huabio, China | 1：2000 |
| Hif1α | Rabbit | A14877 | Abclonal, China | 1：400 |
| Fis1 | Rabbit | YN5292 | immunoway，USA | 1:2000 |
| Omi1 | Rabbit | ET1609-73 | Huabio, China | 1：2000 |
| Pink | Rabbit | ER1706-27 | Huabio, China | 1：1000 |
| Era | Rabbit | HA721140 | Huabio, China | 1：1000 |
| Yy1 | Rabbit | ET1605 | Huabio, China | 1：200 |
| Tomm20 | Rabbit | YM8164 | immunoway，USA | 1:500 |

Table S4 Real-time PCR primer sequences

| Target gene | Primer sequence forward (5’-3’) | Primer sequence reverse (5’-3’) | Accession number |
| --- | --- | --- | --- |
| *β-actin* | CGTGACATCAAGGAGAAG | GAGTTGAAGGTGGTCTCAT | M25013 |
| *nrf1* | CAACAGCCACACACAGCATT | TCCACCTCTCCATCCGTCA | JF939202 |
| *pgc1α* | AAAGCCAGGGAAGCCAAGAG | ATGATGGGGAGGCAGAGGAT | JN195739 |
| *stra6* | AGTGGTAAACCCAAGTGTGTGA | TCTGCAGGGCAAACTTCCTC | XM_051884399.1 |
| *rbp4* | GGAACCTGCTTGGACGGTTA | ACAGAAACCAGTGTGTGCGA | XM_051914319.1 |
| *atf4* | TTCGGCCAACACCTTAGACC | CTTGCCTCATCTTTCGGGGT | AY437846 |
| *ttr* | GGGATTCCCGCTGGAAATGT | GATCAAGTTGTGCACCTCGC | XM_051874080.1 |
| *hsp60* | GCAGTCAAGGCTCCAGGATT | TAGCCTCGTCACCAAACACC | XM_051905556.1 |
| *akt* | CCTGGTGATGAAGGAGCTGA | CTGTCAGAGAGCCTCCAGCA | KY763985 |
| *tfb2m* | GATGTCCCGGTGAAGGTTGT | CCACACGTCCGTAGTGGAAA | XM_051867666.1 |
| *sdha* | CCCCACCAACTACAAGGGAC | AGATCCAGCAGTGAGTTGGC | XM_051871968.1 |
| *atp5a1* | ATCCGGGTCTGTTGTTGACC | GGCAGTGTGCAGGTTCTTTG | XM_051877121.1 |
| *mfn1* | AGTTTGGATGCTGGCTGTCT | GCTTTGAGAGGCGTTCGTTG | CI_GC_23762 |
| *mfn2* | AAAGTGGCAGGGATTGGGG | CGTAGGAAGCAGTTGGTGGT | CI_GC_11929 |
| *dnm1l* | AAACGTAGACCCGGAGGACA | TCCTTTGTAAAGGCGCCCA | XM_051883178.1 |
| *becline1* | ATGGTGGCCTTCCTTGACTG | TGCCACCTGTGTCCTCAATC | XM_051915186.1 |
| *parkin* | ATGGACTTGGCTGTGGGTTT | CACGGGAACTTGGCATTTGG | MZ358120 |
| *bnip3* | CGCTAGACTTACGGGCACAT | TCCACTCGTCCCTTCCTCTT | XM_051913819.1 |
| *p62* | GATGGGGTTGGCTCTTGTGA | GCGAATGAGAACCCAGGGAA | XM_051859922.1 |
| *sirt3* | ATGGACTAGAACGAATGGCTG | CTCTCCCTTGTAATCCCTTCG | XM_051901483.1 |
| *foxo3a* | GCTGCGTAGTGATCCCATGATGTC | GGAGACTGTTGGAGATGCTGCTTC | (Zhu et al, 2024) |

**References:**

Resources N. Nutrient requirements of fish and shrimp. nutrient requirements of fish & shrimp 2011: National academies press.

Zhou, Y., Wu, P., Jiang, W., Liu, Y., Peng, Y., Kuang, S., Tang, L., Li, S., Feng, L., Zhou, X., 2023. Dietary cinnamaldehyde improves muscle protein content by promoting muscle fiber growth via PTP1B/IGF1/PI3K/AKTs-TOR/FOXO3a signaling pathway in grass carp (*Ctenopharyngodon idella*). Food Chemistry. 399**,** 133799.
