# Supplementary material for: Exploring new benefits of vitamin A: alleviating hypoxia-induced mitochondrial stress and mitophagy in the gills of adult grass carp (Ctenopharyngodon idellus)
Source: J Anim Sci Biotechnol. 2025 Dec 16;16:172. doi: 10.1186/s40104-025-01309-3 (PMC12706901; doi:10.1186/s40104-025-01309-3)

Supplementary file (uncropped protein band)

Gapdh in gill

From left to right are: Normoxia 375, 862, 1614, 2099, 2786, 3118 IU/kg; Hypoxia 375, 862, 1614, 2099, 2786, 3118 IU/kg.

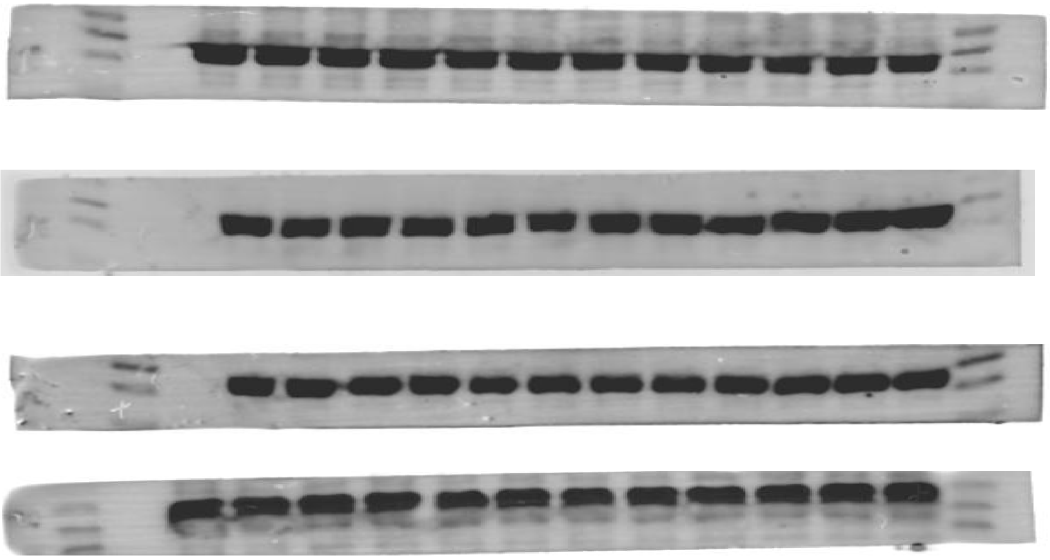

Atf5 in gill

From left to right are: Normoxia 3118, 2786, 2099, 1614, 862, 375 IU/kg; Hypoxia 375, 862, 1614, 2099, 2786, 3118 IU/kg.

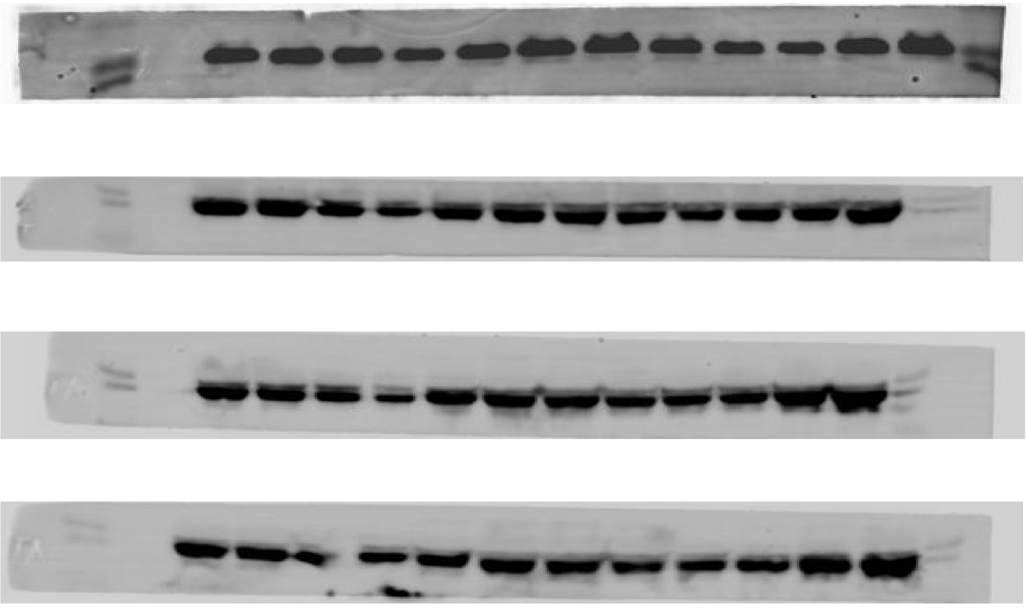

Crbp1 in gill

From left to right are: Normoxia 375, 862, 1614, 2099, 2786, 3118 IU/kg; Hypoxia 375, 862, 1614, 2099, 2786, 3118 IU/kg.

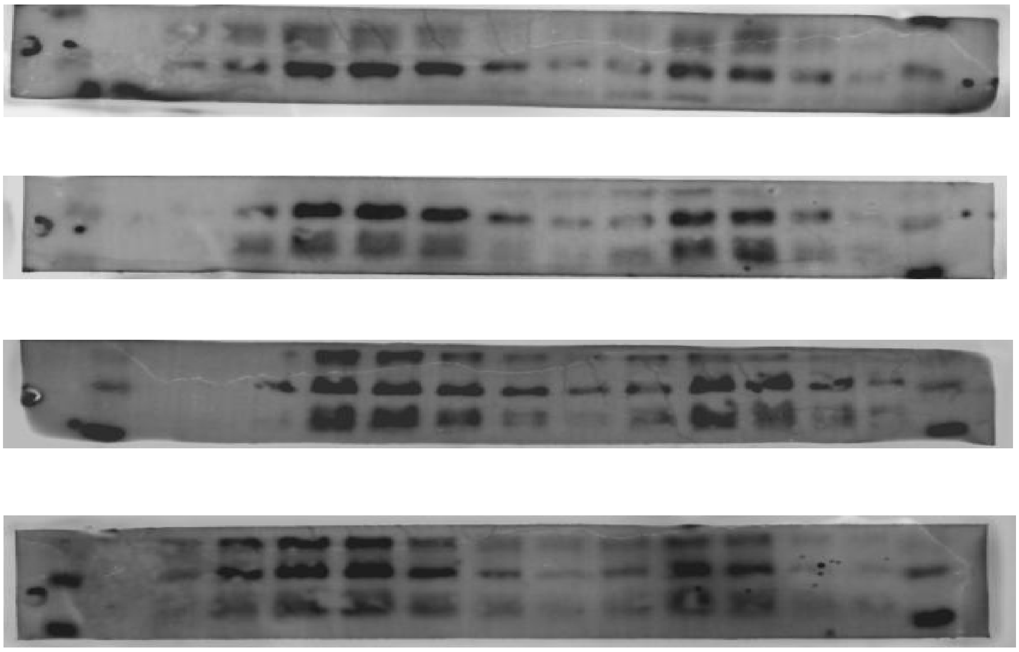

Pink

From left to right are: Normoxia 3118, 2786, 2099, 1614, 862, 375 IU/kg; Hypoxia 3118, 2786, 2099, 1614, 862, 375 IU/kg.

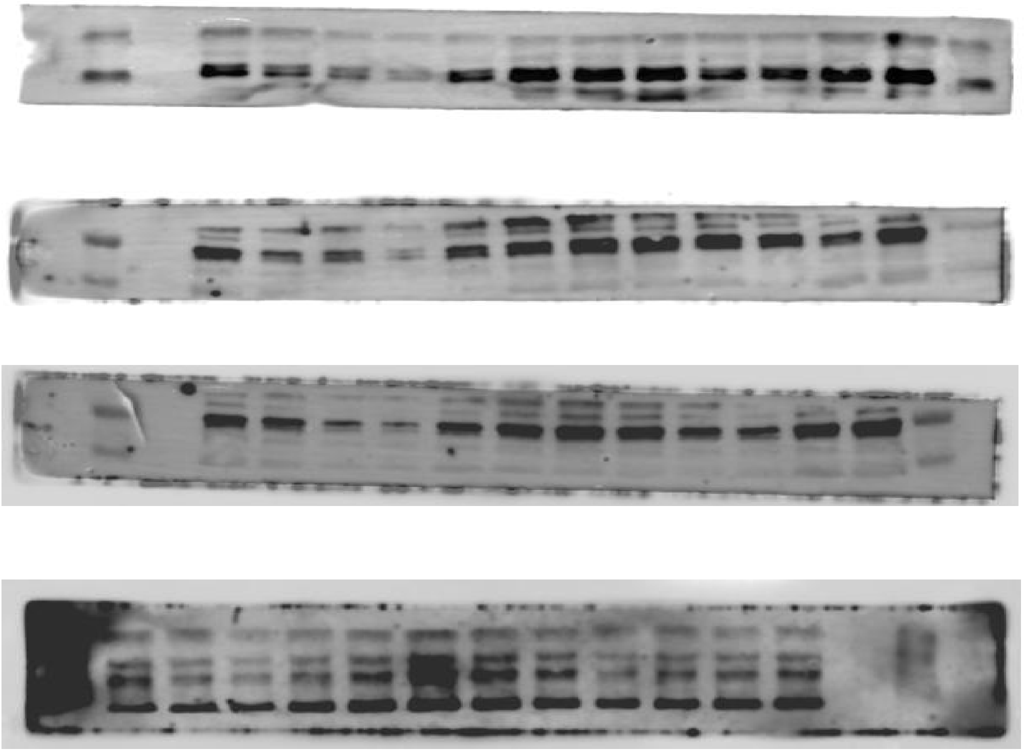

Drp1

From left to right are: Normoxia 375, 862, 1614, 2099, 2786, 3118 IU/kg; Hypoxia 375, 862, 1614, 2099, 2786, 3118 IU/kg.

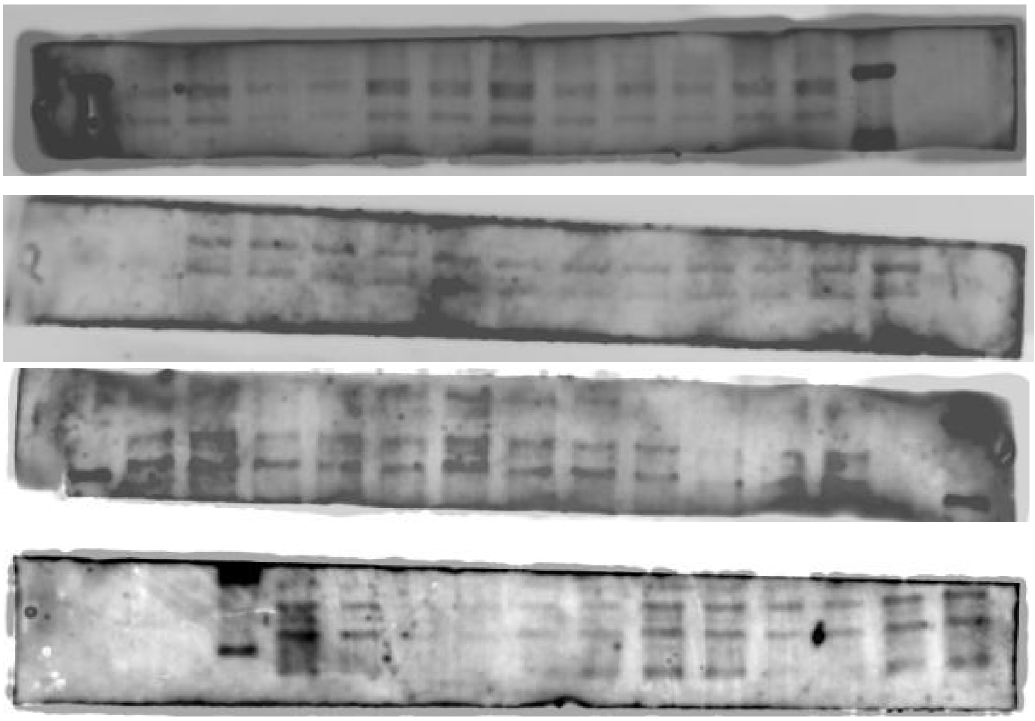

Fis1

From left to right are: Normoxia 375, 862, 1614, 2099, 2786, 3118 IU/kg; Hypoxia 375, 862, 1614, 2099, 2786, 3118 IU/kg.

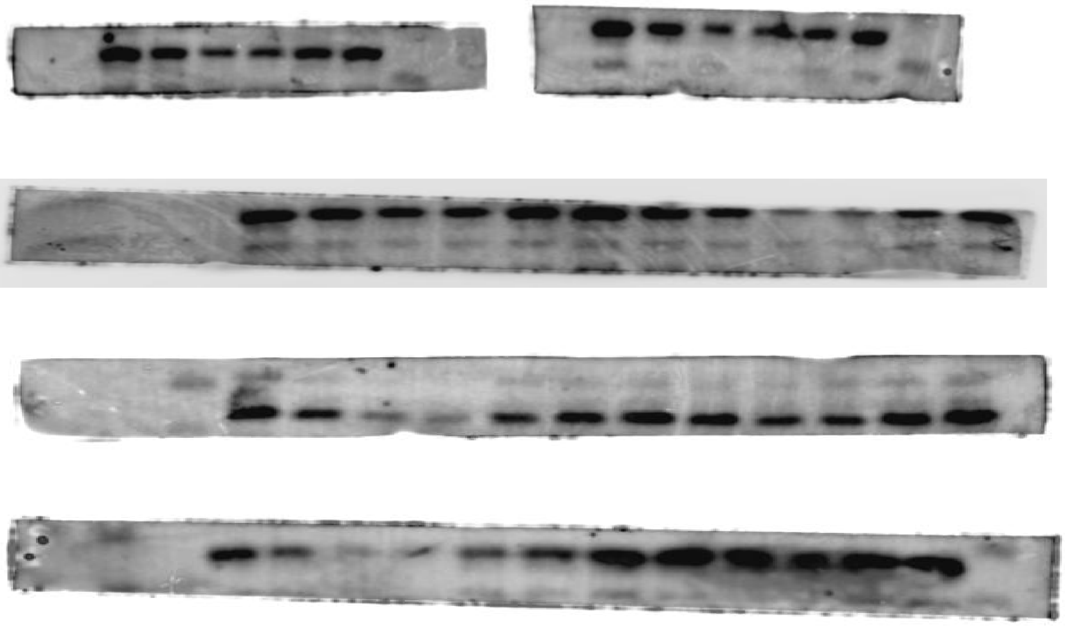

Hif1a

From left to right are: Normoxia 375, 862, 1614, 2099, 2786, 3118 IU/kg; Hypoxia 375, 862, 1614, 2099, 2786, 3118 IU/kg.

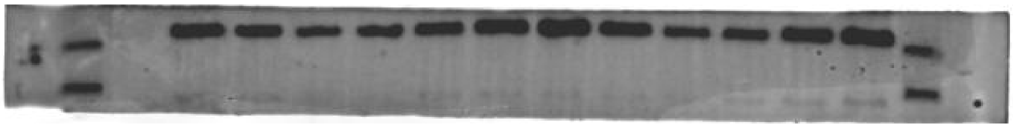

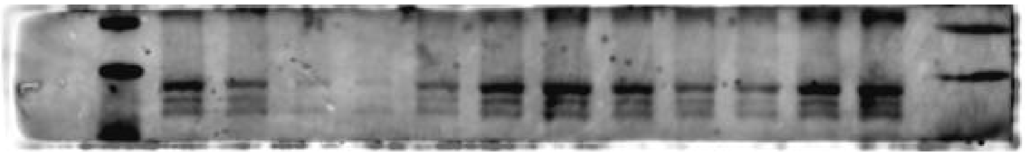

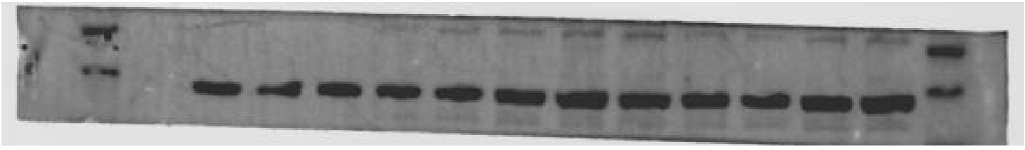

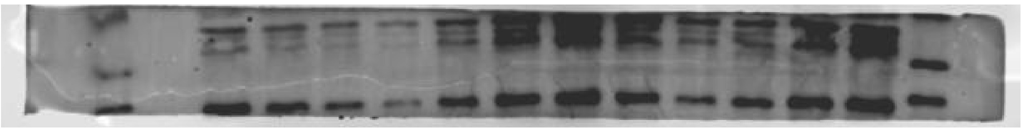

Lc3

From left to right are: Normoxia 375, 862, 1614, 2099, 2786, 3118 IU/kg; Hypoxia 375, 862, 1614, 2099, 2786, 3118 IU/kg.

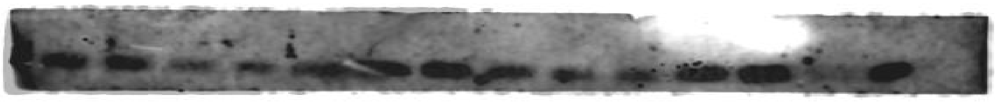

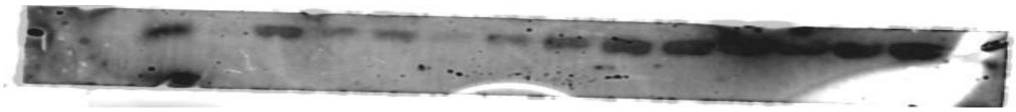

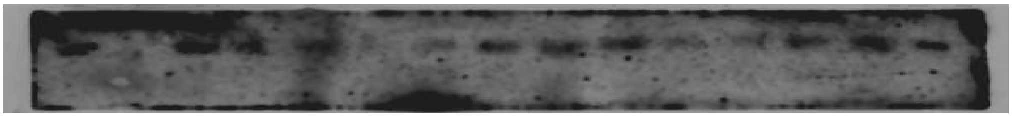

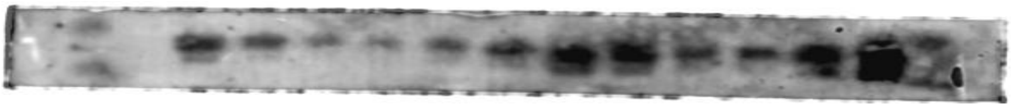

Fgf21

From left to right are: Normoxia 375, 862, 1614, 2099, 2786, 3118 IU/kg; Hypoxia 375, 862, 1614, 2099, 2786, 3118 IU/kg.

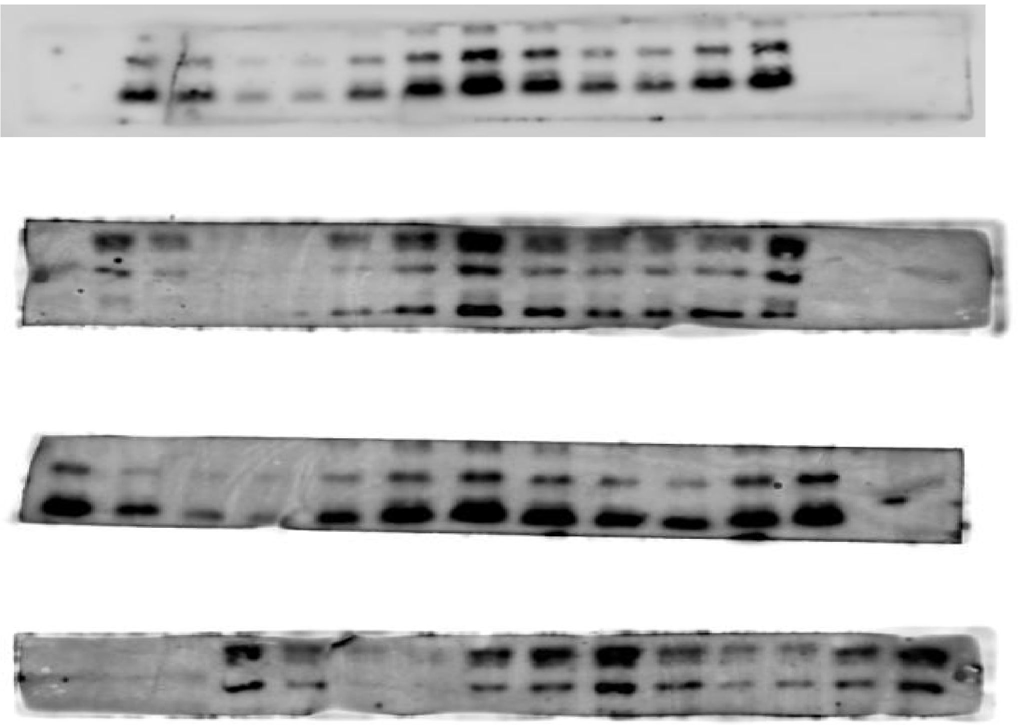

Omi1

From left to right are: Normoxia 3118, 2786, 2099, 1614, 862, 375 IU/kg; Hypoxia 3118, 2786, 2099, 1614, 862, 375 IU/kg.

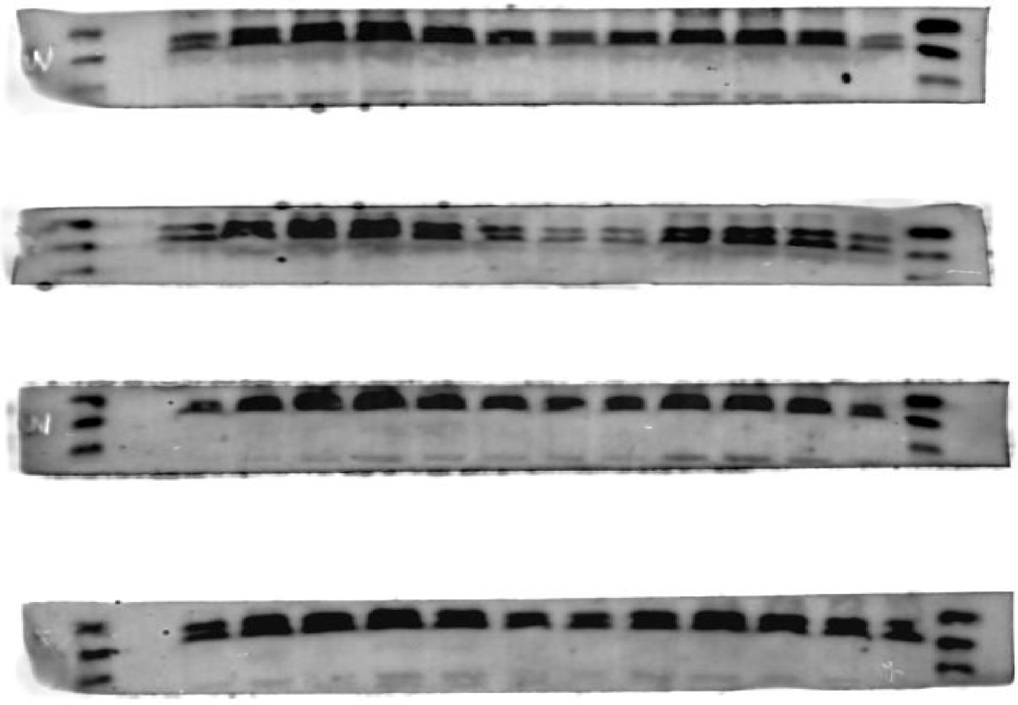

Opa1

From left to right are: Normoxia 375, 862, 1614, 2099, 2786, 3118 IU/kg; Hypoxia 375, 862, 1614, 2099, 2786, 3118 IU/kg.

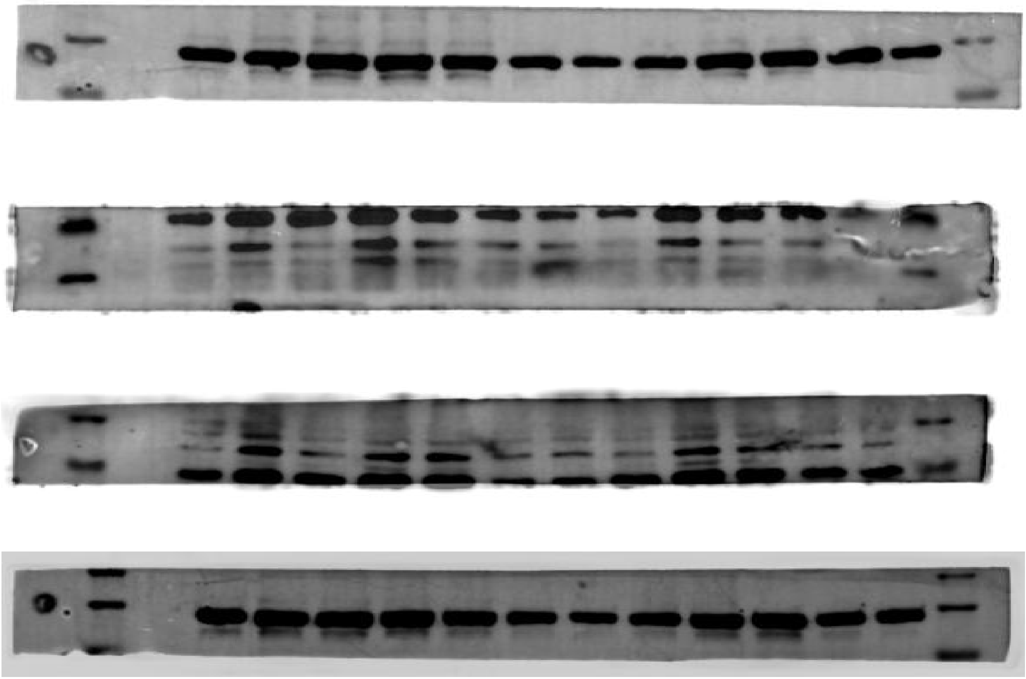

Supplement: Supplementary file 2 — Additional file 2: The original gel and blot images. [file 40104_2025_1309_MOESM2_ESM.pdf]
